# Short isocapnic hyperoxia affects indices of vascular remodeling and intercellular adhesion molecules in healthy men

**DOI:** 10.1590/1414-431X2022e12110

**Published:** 2022-06-13

**Authors:** V.P. Garcia, J.D. Mattos, J. Mentzinger, P.E.C. Leite, H.N.M. Rocha, M.O. Campos, M.P. Rocha, D.E. Mansur, N.H. Secher, A.C.L. Nóbrega, I.A. Fernandes, N.G. Rocha

**Affiliations:** 1 Laboratório de Ciências do Exercício, Departamento de Fisiologia e Farmacologia, Universidade Federal Fluminense, Niterói, RJ, Brasil; 2 Laboratório de Bioengenharia e Toxicologia in Vitro, Instituto Nacional de Qualidade e Tecnologia Metrológica, Duque de Caxias, RJ, Brasil; 3 Department of Anesthesia, Rigshospitalet, Institute of Clinical Medicine, University of Copenhagen, Copenhagen, Denmark; 4 NeuroVASQ - Laboratório de Fisiologia Integrativa, Faculdade de Educação Física, Universidade de Brasília, Brasília, DF, Brasil

**Keywords:** Endothelium, Hyperoxia, ICAM-1, Metalloproteinase-9, Vascular remodeling

## Abstract

In preparation for tracheal intubation during induction of anesthesia, the patient may be ventilated with 100% oxygen. To investigate the impact of acute isocapnic hyperoxia on endothelial activation and vascular remodeling, ten healthy young men (24±3 years) were exposed to 5-min normoxia (21% O_2_) and 10-min hyperoxia trials (100% O_2_). During hyperoxia, intercellular adhesion molecules (ICAM-1) (hyperoxia: 4.16±0.85 *vs* normoxia: 3.51±0.84 ng/mL, P=0.04) and tissue inhibitor matrix metalloproteinase 1 (TIMP-1) (hyperoxia: 8.40±3.84 *vs* normoxia: 5.73±2.15 pg/mL, P=0.04) increased, whereas matrix metalloproteinase (MMP-9) activity (hyperoxia: 0.53±0.11 *vs* normoxia: 0.68±0.18 A.U., P=0.03) decreased compared to the normoxia trial. We concluded that even short exposure to 100% oxygen may affect endothelial activation and vascular remodeling.

## Introduction

Prolonged exposure to high oxygen levels (hyperoxia) has been related to lung injury, increased myocardial infarct size, and mortality (1,2), whereas the effects of short exposure to hyperoxia are not fully understood. In preparation for tracheal intubation during induction of anesthesia, the patient may be ventilated with 100% oxygen and the vascular endothelium may be challenged by oxygen toxicity. *In vitro*, hyperoxia seems to provoke an extended pro-atherogenic endothelial cell phenotype by inducing oxidative stress and endothelial cell inflammation (3).

*In vitro* exposure of endothelial cells to hyperoxia leads to a pro-inflammatory state with an increase in endothelial expression of cell adhesion molecules (CAMs) (4). CAMs are involved in the binding of cells with other cells and with the extracellular matrix (ECM), mediating leukocyte adhesion to the vascular endothelium, which can increase the inflammatory response and vascular permeability and is associated with the early stages of atherosclerosis (3). Furthermore, hyperoxia is an important modulator of ECM, resulting in vascular remodeling (5,6). The regulation of ECM turnover is defined by the balance between the activity of matrix metalloproteinase (MMPs), zinc-dependent endopeptidases with the ability to degrade components of ECM, and their tissue inhibitors of matrix metalloproteinase (TIMPs) (7). Increased MMPs activity results in elastin degradation leading to decreased elasticity, and reduced TIMPs levels provoke accumulation of collagen (8). Among all MMPs, the matrix metalloproteinase-9 (MMP-9) or gelatinase B seems to be the main MMP responsible for cardiovascular remodeling in humans (9) and its ability to degrade components of the ECM has been associated to structural and functional vascular alterations in both physiological and pathological conditions (8,9). Taken together, the potential acute effects of hyperoxia on endothelial activation and vascular remodeling need to be addressed *in vivo*.

In addition, hyperoxia induces hyperventilation accompanied by reduced arterial carbon dioxide tension (here expressed as the end-tidal value, PetCO_2_), and CO_2_ influences the release of nitric oxide (NO) from endothelial cells (10,11). Since NO affects adherence of leukocytes to the endothelium by inhibition of intercellular adhesion molecue-1 (ICAM-1) and vascular cell adhesion protein-1 (VCAM-1) expression (12), isocapnic hyperoxia could control the CO_2_ confounding effects. This study evaluated the impact of short-term exposure to isocapnic hyperoxia on cell adhesion molecules and vascular remodeling in healthy men. We hypothesized that exposure to hyperoxia provokes an increase in CAM expression and MMP-9 activity along with reducing TIMP-1 levels in healthy men.

## Material and Methods

### Ethical approval

After verbal explanation, all subjects signed a consent form with a detailed explanation of the experimental procedures before participation in the study. The study protocol was approved by the Ethical Committee of Fluminense Federal University (CAAE: 57077316.1.0000.5243) according to the standards set by the latest revision of the Declaration of Helsinki.

### Subjects

The subjects were recruited through advertisements at the university campus and in local newspapers. Fourteen men were invited to join the study, of which 10 individuals (24±3 years; body mass index of 24±2 kg/m^2^; systolic/diastolic pressure 124±9/71±6 mmHg) were eligible to take part. Inclusion criteria were no history of smoking, no regular physical exercise (<150 min per week of moderate-intensity cardiorespiratory exercise training), no cardiovascular, metabolic, or neurological diseases, and no current pharmacological therapy or nutritional supplementation.

### Instrumentation

Respiratory rate and depth were registered using a piezoelectric respiratory belt transducer (MLT1132, ADInstruments, Australia) positioned in the upper or lower quadrant of the abdomen. Oxygen saturation was quantified on the earlobe (Oximeter Pod, ADInstruments). Ventilation (VE), PetO_2_, and PetCO_2_ were continuously assessed using a breath-by-breath circuit while the subjects breathed through a face mask or a mouthpiece with a nose clip connected to a gas analyzer system (Ultima CPX; Medgraphics, USA).

### Experimental setup

The experimental sessions were in the morning and at least 48 h after a hyperoxia familiarization session. The subjects were instructed to abstain from caffeine, alcohol, and intense exercise for 24 h before the experimental session. After instrumentation, subjects remained in supine rest for 15 min in a dark, temperature-controlled (21-23°C) quiet room. Respiratory rate, VE, tidal volume, PetO_2_, and PetCO_2_ were monitored for 10 min while the subjects breathed normoxic gas (21% O_2_ and 79% N_2_) to establish the target PetCO_2_ for the normoxic and hyperoxic trials. Subsequently, the subjects breathed at a rate of 20 cycles per minute for a 5-min normoxic trial and a 10-min hyperoxic (100% O_2_) trial. The isocapnic breathing pattern was maintained using a metronome, verbal instructions to control breathing amplitude, and a rebreathing circuit to prevent poikilocapnic hyperoxia-induced hyperventilation (13). At the last minute of each trial, blood was taken from the antecubital vein (and replaced with NaCl 0.9%) to quantify cell adhesion molecules and vascular remodeling.

### Matrix metalloproteinase-9 activity

The MMP-9 activity, a proxy of extracellular matrix turnover and vascular remodeling, was assessed by zymography in plasma samples (14). The gelatinolytic activity was detected as unstained bands against the dark blue background of the Coomassie blue-stained gelatin using an Epson digital scanner. An internal standard (control plasma sample) and a protein molecular weight marker (161-0375, Bio-Rad, USA) were used to allow inter-gel analysis and comparison. Scion Image software (Scion Corporation, USA) was used to quantify band intensities. The active form of MMP-9 was identified as a band at 87 kDa (see Supplementary Figure S1).

### Tissue inhibitor of metalloproteinase concentration

Plasma TIMP-1 was measured by enzyme-linked immunosorbent assay (ELISA) using a commercial kit (Human TIMP-1 Tissue Inhibitors of Metalloproteinase 1, Elabscience^®^, USA) following the manufacturer's instructions. Plasma samples were not diluted and absorbance was read at 450 nm.

### ICAM-1, VCAM-1, and P-selectin concentration

Cell adhesion molecules (ICAM-1, VCAM-1, and P-selectin) were measured in plasma by a commercial kit (Human Cardiovascular Disease Magnetic Bead Panel 2, Millipore Sigma, USA) according to manufacturer's instructions. Quantification of the magnetic beads was performed with a BioPlex MAGPIX system (Biorad, US) and results were analyzed using Xponent software (Luminexcorp, USA). The analyses of the concentration of cell adhesion molecules involved only eight subjects because of technical problems with the plasma samples.

### Statistics

Data are reported as means±SD. Normal distribution and homogeneity of variance were verified using the Shapiro-Wilk test and Levene's test, respectively. When appropriate, a two-tailed paired Student's *t*-test or Wilcoxon signed rank test was used to compare the variables between normoxic and hyperoxic trials. The index of net MMP-9 activity was calculated as the ratio between MMP-9 activity and TIMP-1 concentration. The effect size of hyperoxia was calculated using Cohen's d. A sample size of 8 subjects was considered necessary to detect a 5% difference between trials to establish a P-value of 0.05 and power of 0.80. Significance was accepted at the 0.05 level and all analyses were carried out with Statistica software (StatSoft Inc., USA).

## Results

Table 1 shows the respiratory responses to normoxia and hyperoxia. As expected, hyperoxia evoked an increase in PetO_2_ (P=0.01 *vs* normoxia) and O_2_ saturation (P=0.008 *vs* normoxia). As intended, there was no change in PetCO_2_ or VE during hyperoxia (P>0.05).

**Table 1:** Respiratory variables at normoxia and during hyperoxia.

	Normoxia (21% O_2_)	Hyperoxia (100% O_2_)	P-value
PetO_2_ (mmHg)	98.8±3.9	480.2±4.3	0.01
PetCO_2_ (mmHg)	40.6±1.0	40.4±1.0	0.80
O_2_ saturation (%)	98.8±0.3	100±0.0	0.01
VE (L/min)	11.6±4.5	11.5±3.5	0.84

Data are reported as means±SD. Two-tailed paired Student's *t*-test. PetO_2_: end-tidal oxygen arterial pressure; PetCO_2_: end-tidal carbon dioxide arterial pressure; VE: ventilation.

**Figure 1:**
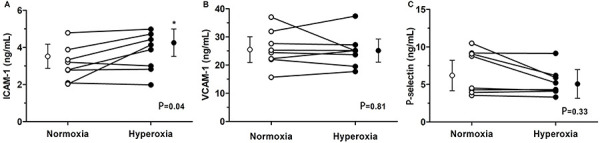
Venous plasma levels of ICAM-1 (**A**), VCAM-1 (**B**), and P-selectin (**C**) in healthy men (n=8) at normoxia (21% O_2_) and during hyperoxia (100% O_2_). Data are reported as means±SD. *P*<*0.05 *vs* normoxia (Wilcoxon signed-rank test). ICAM-1: intercellular adhesion molecule 1; VCAM-1: vascular cell adhesion molecule 1.

The effect of hyperoxia on cell adhesion molecules is shown in Figure 1. During hyperoxia, the level of ICAM-1 increased (∼18%) compared to the normoxia trial (hyperoxia: 4.16±0.85 *vs* normoxia: 3.51±0.84 ng/mL, P=0.04; Cohen's d=0.91). In contrast, hyperoxia did not change the level of VCAM-1 (normoxia: 26.62±8.78 *vs* hyperoxia: 25.79±6.78 pg/mL, P=0.81) or P-selectin (normoxia: 6.07±2.92 *vs* hyperoxia: 5.01±1.72 pg/mL, P=0.33).

**Figure 2:**
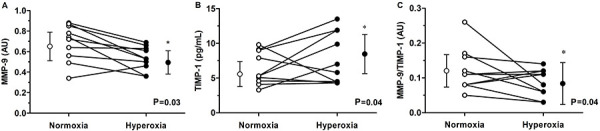
Venous plasma MMP-9 activity (**A**), TIMP-1 levels (**B**), and net MMP-9 activity (**C**) in healthy men (n=10) exposed to normoxia (21% O_2_) and hyperoxia (100% O_2_). Data are reported as means±SD. *P*<*0.05 *vs* normoxia (Wilcoxon signed-rank test). MMP-9, matrix metalloproteinase-9; TIMP-1: tissue inhibitor of metalloproteinase 1.

The effect of hyperoxia on MMP-9, TIMP-1, and net MMP-9 activity are shown in Figure 2. During hyperoxia, the activity of MMP-9 decreased (∼22%) compared to normoxia trial (hyperoxia: 0.53±0.11 *vs* normoxia: 0.68±0.18 A.U., P=0.03; Cohen's d=1). Along with MMP-9 activity, hyperoxia evoked a greater increase (∼46%) in TIMP-1 levels (hyperoxia: 8.40±3.84 *vs* normoxia: 5.73±2.15 pg/mL, P=0.04; Cohen's d=0.73). The MMP-9/TIMP-1 ratio was low (∼41%) in response to hyperoxia (hyperoxia: 0.07±0.04 *vs* normoxia: 0.12±0.06 A.U., P=0.04).

## Discussion

**Figure 3:**
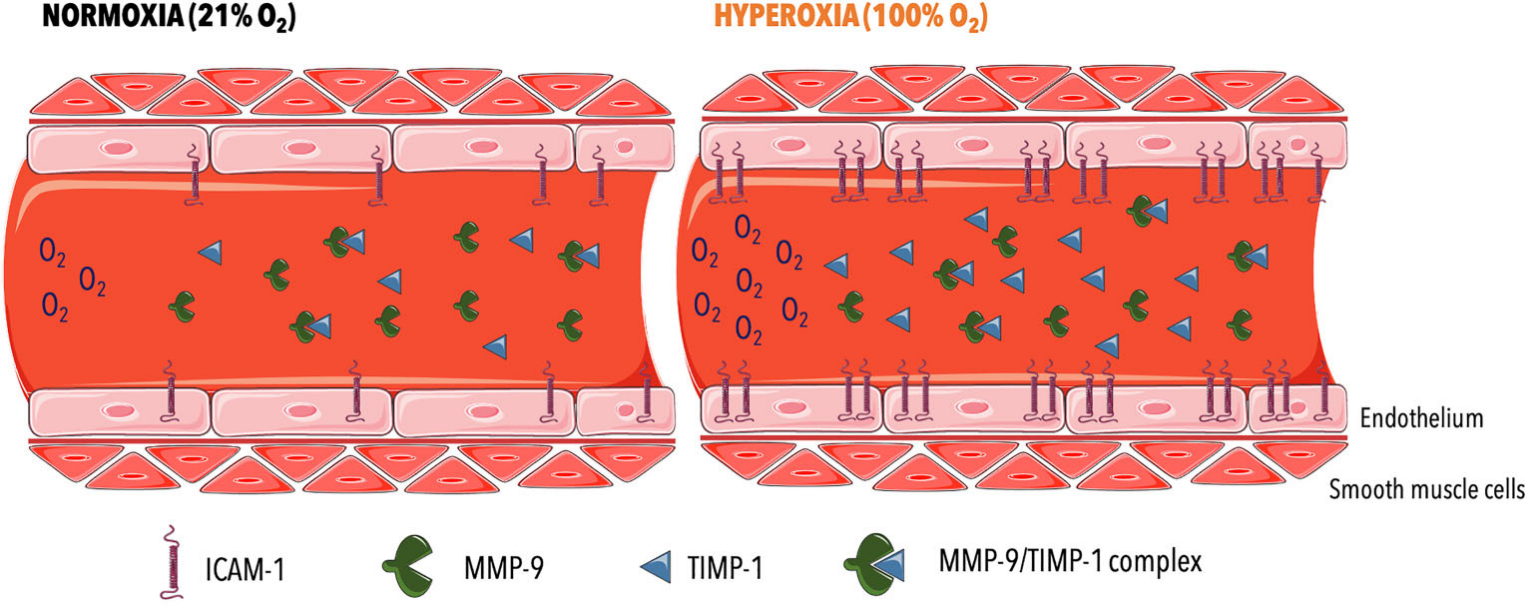
Hyperoxia induces activation of the endothelium via increased ICAM-1 expression and stimulates the extracellular matrix dysregulation characterized by increased TIMP-1 levels and reduced MMP-9 activity. ICAM-1: intercellular adhesion molecule 1; MMP-9: matrix metalloproteinase-9; TIMP-1: tissue inhibitor of metalloproteinase 1.

To secure tissue oxygenation during a difficult tracheal intubation, the patient may be exposed to “pre-oxygenation” using 100% oxygen, often changed to inhalation of, e.g. 30% oxygen and mild positive pressure ventilation in order to prevent alveolar collapse once the tube is in place (15). This study found that the ICAM-1 level is increased in response to even 10 min of hyperoxia without affecting VCAM-1 and P-selectin levels. In contrast to our hypothesis, exposure to hyperoxia reduced MMP-9 activity and increased TIMP-1 levels in healthy men (Figure 3), and these findings likely contributed to reduction of the MMP-9/TIMP-1 ratio.

Endothelial activation is a term used to characterize an increase in adhesion molecules on the endothelial surfaces including ICAM-1, VCAM-1, and P-selectin. Hyperoxia promoted an increase in ICAM-1 levels without affecting VCAM-1 or P-selectin levels. Accordingly, ICAM-1 is upregulated in response to hyperoxia (90% O_2_-5% CO_2_, 48-72 h) in both cultured human umbilical vein endothelial cells (HUVEC) and pulmonary endothelial cells (HPAEC) (3,4). Also, E-selectin and P-selectin levels did not change after exposure of endothelial cells to hyperoxia (4). Taken together, findings indicate that hyperoxia selectively upregulates the expression of ICAM-1 in healthy men, indicating endothelial activation.

Healthy men presented a decreased MMP-9 activity and increased TIMP-1 levels, which was consistent with a reduced MMP-9/TIMP-1 ratio. Although some *in vitro* studies and studies with animal models have observed an increase in MMP-9 concentration (50%) and activity (85%) after exposure to hyperoxia (6,16); others demonstrated that hyperoxia (>95% O_2_) provokes MMP-9 down-regulation (5,17) and increases levels of TIMP-1 (5,18). It is believed that hyperoxia regulation depends on time exposure, i.e., the up-regulation of MMP activity is associated with prolonged exposure (>24 h), whereas down-regulation seems to be associated with a short exposure (<12 h) (17). Therefore, these findings represent evidence that hyperoxia triggered disturbances in vascular remodeling in healthy men.

Finally, it is conceivable that hyperoxia may exacerbate endothelial dysfunction in subjects with cardiovascular risk factors and cardiovascular disease, leading to worse cardiovascular outcomes. Furthermore, it is important to highlight that oxygen is the most common treatment strategy used in hospitalized patients with COVID-19. Given the significantly prolonged exposure to hyperoxia in intubated COVID-19 patients, we speculate that hyperoxia would provoke a fierce vascular response, resulting in increased inflammation, oxidative stress, expression of adhesion molecules, and vascular remodeling (19). However, the effect of hyperoxia on endothelium in patients with COVID-19 needs to be investigated.

Some limitations should be considered. First, the effect of hyperoxia on endothelium needs to be investigated under a poikilocapnic condition. Second, the trial order (normoxia and isocapnic hyperoxia) was not randomized. Nevertheless, we believe that this methodological concern does not invalidate our conclusions. Third, the activity of other metalloproteinases related to cardiovascular remodeling was not measured (i.e., MMP-2 and ADAM17). Finally, the present study was conducted in healthy men because women seem to be less susceptible to changes in cardiovascular physiology under hyperoxia (20). However, further studies are necessary to understand the hyperoxic effects on cell adhesion molecules and vascular remodeling in women, older adults, and in chronic diseases.

In conclusion, acute isocapnic hyperoxia in healthy men induced endothelial activation through increasing ICAM-1 and altered vascular remodeling by decreasing the MMP-9 and TIMP-1 ratio. Thus, we suggest that even short exposure to 100% oxygen affects endothelial adhesion and vascular remodeling factors, and that pre-oxygenation before anesthesia should be carried out with an oxygen level lower than 100%, significantly reducing these effects on vasculature.

## Supplementary Material

Click to view [pdf].
